# Optimisation of complex health interventions prior to a randomised controlled trial: a scoping review of strategies used

**DOI:** 10.1186/s40814-016-0058-y

**Published:** 2016-03-15

**Authors:** Sara Levati, Pauline Campbell, Rachael Frost, Nadine Dougall, Mary Wells, Cam Donaldson, Suzanne Hagen

**Affiliations:** 1 NMAHP Research Unit, Glasgow Caledonian University, Level 6, Govan Mbeki Building 70 Cowcaddens Road, Glasgow, G4 0BA Scotland; 2 NMAHP Research Unit, University of Stirling, Unit 13 Scion House, Innovation Park, Stirling, FK9 4NF UK; 3 Yunus Centre for Social Business and Health, Glasgow Caledonian University, M201, George Moore Building 70 Cowcaddens Road, Glasgow, G4 0BA Scotland

**Keywords:** Complex interventions, Intervention development, Pre-trial, Optimisation, Modelling, Scoping review, Effectiveness, Acceptability

## Abstract

**Background:**

Many complex intervention trials fail to show an intervention effect. Although this may be due to genuine ineffectiveness, it may also be the result of sub-optimal intervention design, implementation failure or a combination of these. Given current financial constraints and the pressure to reduce waste and increase value in health services research, pre-trial strategies are needed to reduce the likelihood of design or implementation failure and to maximise the intervention’s potential for effectiveness. In this scoping review, we aimed to identify and synthesise the available evidence relating to the strategies and methods used to ‘optimise’ complex interventions at the pre-trial stage.

**Methods:**

We searched MEDLINE, CINAHL, AMED, PsycINFO and ProQuest Nursing & Allied Health Source for papers published between January 2000 and March 2015. We included intervention development and optimisation studies that explored potential intervention weaknesses and limitations before moving to a definitive randomised controlled trial (RCT). Two reviewers independently applied selection criteria and systematically extracted information relating to the following: study characteristics; intervention under development; framework used to guide the development process; areas of focus of the optimisation process, methods used and outcomes of the optimisation process. Data were tabulated and summarised in a narrative format.

**Results:**

We screened 3968 titles and 231 abstracts for eligibility. Eighty-nine full-text papers were retrieved; 27 studies met our selection criteria. Optimisation strategies were used for a range of reasons: to explore the feasibility and acceptability of the intervention to patients and healthcare professionals; to estimate the effectiveness and cost-effectiveness of different combinations of intervention components; and to identify potential barriers to implementation. Methods varied widely across studies, from interviews and focus groups to economic modelling and probability analysis.

**Conclusions:**

The review identifies a range of optimisation strategies currently used. Although a preliminary classification of these strategies can be proposed, a series of questions remain as to which methods to use for different interventions and how to determine when the intervention is ready or ‘optimised enough’ to be tested in a RCT. Future research should explore potential answers to the questions raised, to guide researchers in the development and evaluation of more effective interventions.

## Background

Complex health interventions (CHIs) are defined as multicomponent interventions in which individual, collective and organisational elements act both independently and interdependently [1]. Interactions between intervention components and their effects on outcomes are not always linear or obvious, and they are influenced by several factors [2]. These include, for example, the number of interacting components, the intensity of behaviour change required by those delivering or receiving the intervention, the number of groups or organisational levels targeted by the intervention and the complexity of outcomes, as well as the context in which interventions are implemented [3–5]. This results in considerable challenges to the evaluation of CHIs, which in turn requires substantial resources.

Randomised controlled trials (RCTs) are historically recognised as the ‘gold standard’ methodology in the evaluation of interventions and they have a long record of successful application in determining a causal relationship between an intervention and its putative outcomes [6]. However, in the case of an intervention that does not influence the outcomes as expected, trials often fail to detect or report whether the lack of intervention effect is due to sub-optimal intervention design, implementation failure or genuine ineffectiveness [7, 8].

As Sermeus states, the increasing number of components that characterise interventions leads to them being even more complex, less understood and much harder to implement [9]. This raises two specific questions; the first one, how to understand if the intervention works as predicted, and the second when it is time to move to the evaluation phase and test the intervention in a full-scale RCT. Methods to improve intervention design, reduce implementation failure and enhance trial processes have developed considerably over the past 15 years and several frameworks and practical guidelines have been issued. In this paper, we focus on probably the least explored and understood process related to the development of complex interventions: the *optimisation* of the intervention under development prior to a full-scale RCT. In the late 2000s, Collins and colleagues introduced the multiphase optimisation strategy (MOST) framework—a strategy for developing and *optimising* behaviour interventions. The element of focus of this framework is the role of the different intervention components and their contribution to the overall success of the intervention, as complex interventions may contain inactive components [10]. The framework proposes to adopt a programmatic and sequenced experimental approach that can efficiently and systematically identify the most promising components, in order to assemble these in an *optimised* version of the intervention, which is finally tested in a RCT. While the MOST framework has some conceptual roots in the phased approach to intervention development and evaluation proposed by the Medical Research Council (MRC), it draws attention to the importance of optimising complex interventions—where optimised interventions are defined as ‘the most effective intervention given certain constraints’, such as for example the resources available for the intervention or the time available for the delivery (e.g. intervention delivered for ≤$500 or for a maximum of 10 h/week per healthcare professional).

**Fig. 1:**
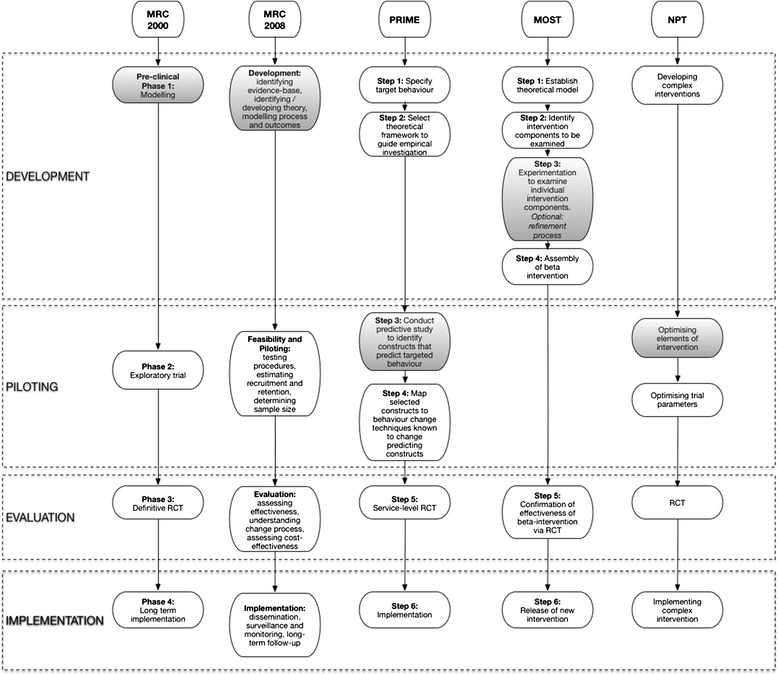
Key phases of the main frameworks that currently provide guidance on conducting pre-trial testing. *Shaded boxes* indicate ‘optimisation’ stages. These frameworks have all been employed, to varying degrees, to define potential limitations and weaknesses of the intervention, in order to refine the intervention before moving to the trial stage.

Alternative approaches to CHI optimisation include strategies proposed by the MRC framework 2000 [1] and 2008 [3], the normalisation process theory (NPT) [11, 12] and the process modelling in implementation research (PRIME) approach [13]. The key stages of each of these frameworks and guidelines are shown in Fig. 1. These guidelines and frameworks acknowledge the need to limit sub-optimal intervention design and implementation failure. As such, they all emphasise the importance of testing the intervention’s potential effect and evaluating how interventions work before embarking on a full-scale RCT (Fig. 1). However, researchers could be forgiven for not knowing which methods to use and when. Existing frameworks differ in the language and terminology used, and there is a lack of clarity over the specific purpose and scope of each proposed stage of work to be conducted before the full-scale RCT [14]. In addition, the different guidelines and frameworks propose a range of methods, from computer simulations and factorial experiments to qualitative studies involving key stakeholders. But evidence to support the use of these methods for particular purposes is lacking, and there is limited guidance on the specific detail of how to plan and design optimisation studies. This leads to confusion about which guideline or framework to follow and which optimisation strategy is likely to be most suitable for the different types of intervention under evaluation.

For the purpose of this review, we have defined optimisation as a process aimed to evaluate or test intervention components and/or drafted interventions in order to identify what works and what does not work within the intervention under design. Thus, the ultimate aim of optimisation processes is to isolate those interventions or intervention components that are more likely to be effective if implemented in a full-scale trial setting. For this reason, we use the term ‘optimisation strategies’ as an umbrella term to encompass a wide range of approaches, such as those referred to above, used to optimise the intervention itself before moving to a full-scale RCT. This means that we are not exploring optimisation strategies aimed to optimise trial parameters, such as recruitment and randomisation processes, in preparation for the main trial. Furthermore, this review focuses exclusively on those strategies adopted before moving to the full-scale RCT stage. As such, optimisation processes may represent a separate stage or can be integrated within the development or pilot and feasibility phase.

This scoping review aimed to explore the strategies and methods currently used by researchers to optimise CHIs before the definitive trial stage so as to understand how, when and why certain strategies might be most usefully applied.

## Methods

### Design

The rapid increase in the amount of primary research available has led to the development of different and new strategies for synthesising evidence in a more effective and rigorous way [15]. Scoping reviews represent a useful and increasingly popular method of collecting and organising important background information on a topic and are described as a process for mapping the existing literature. In 2005, Arksey and O’Malley proposed a framework for conducting scoping reviews, which included the following five iterative stages: (1) identifying the research question(s); (2) identifying relevant studies; (3) study selection; (4) charting the data; (5) collating, summarising and reporting the results [16]. Scoping reviews can be conducted for several reasons, such as to map fields of studies where it is difficult to anticipate the range of material that might be available, to determine the value of undertaking a systematic review and define more precise questions and suitable inclusion criteria, to identify research gaps in the existing literature or to clarify working definitions and/or the conceptual boundaries of a topic. Typically, scoping reviews differ from systematic reviews in several ways, as outlined in Table 1. Scoping reviews, in particular, identify a broader ‘scope’ and research questions with less restrictive inclusion and exclusion criteria, which are determined in an iterative way on the basis of familiarity with the literature [17, 18]. Another important distinction between scoping reviews and systematic reviews is that, unless otherwise specified, a quality assessment of the included studies is generally not performed [19].

**Table 1:** General comparisons between scoping and systematic reviews

Review characteristics	Systematic review	Scoping review
Research question	Typically focused, narrow parameters	Broad
Selection criteria	Pre-defined	Can be developed post hoc using an iterative approach [16, 18]
Quality assessment	Filters applied	Quality filters not often applied in initial stages
Data extraction	Pre-defined, a priori and generally very detailed. Usually well documented in a protocol ahead of the review process	Less structured. Data extraction process typically ‘evolves’ as a part of the scoping review process
Evidence synthesis	Quantitative (sometimes mixed)	Typically qualitative
Other		Often described as a process of mapping existing literature and used to identify gaps in evidence

**Fig. 2:**
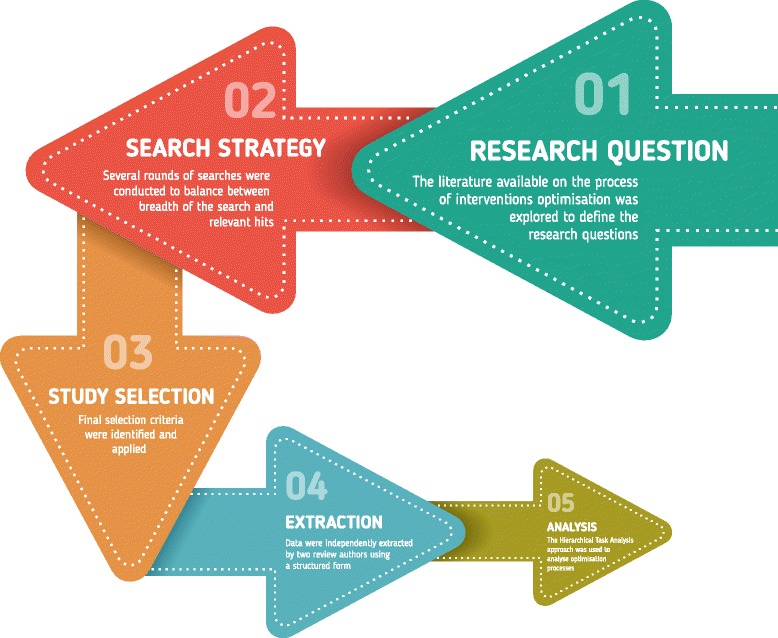
Iterative approach followed to conduct this scoping review

The purpose of this scoping review was to map the literature available on the optimisation of CHIs before moving to an RCT and to identify potential gaps in the current literature. The review followed the iterative stages proposed by Arksey and O’Malley, with each feeding into the next stage (Fig. 2). To add rigour to the review process, a systematic team approach was adopted. Team meetings included iterative discussions surrounding keywords to be searched, inclusion/exclusion criteria and study selection at different stages of the review process. Prior to conducting this scoping review, the literature was searched in order to identify any frameworks and guidelines for researchers that had been published on the development and evaluation of complex health interventions. This enabled us to familiarise ourselves with the different recommended stages of intervention development and the terminology used to refer to optimisation processes. The recent guidance issued by Joanna Briggs Institute [15] for reporting scoping reviews is used here to describe the different criteria and processes adopted in our scoping review.

### Inclusion criteria

For the purpose of this review, we included any type of pre-trial study, such as intervention development studies, pilot and feasibility studies that either explicitly referred to optimisation or described processes that fitted with our definition of optimisation studies. The review used an exploratory approach and it encompassed any type of CHI, including those targeting patients and those designed to address healthcare professionals’ practice. Final selection criteria agreed by the review team at multiple consensus meetings are shown in Table 2.

**Table 2:** Selection criteria agreed during several consensus meetings

	Interventions	Optimisation strategies
Inclusion criteria	Complex health interventions defined as multicomponent interventions [1]	Aimed to test and evaluate the intervention and/or its components in order to define potential limitations and weaknesses before moving to the main evaluation phase
		Optimisation process to be conducted in the pre-trial stage, defined as part of the development stage or part of the pilot or feasibility study in preparation for the main trial
Exclusion criteria	Complementary and alternative medicines	Optimisation process focused on trial parameters
		Conference abstracts and posters
		Study protocols
		Methodological papers with no empirical data reported^a^

This was an iterative process, following the recommendations of Arksey and O’Malley [16]

^a^Methodological papers were excluded but strongly informed the iterative design and the process of this scoping review

### Search strategy

To conduct this scoping review, we searched the following electronic databases, MEDLINE, CINAHL, AMED, The Cochrane Methodology Register and PsycINFO and ProQuest Nursing & Allied Health Source for peer-reviewed publications. We limited our searches to publications in the English language only and those published between 1 January 2000 to 31 March 2015. The search strategy used a combination of key terms related to complex health interventions, keywords related to the overall process of optimising complex interventions, together with framework or guideline-specific keywords used to describe an optimisation process (e.g. ‘modelling’ for the MRC framework, ‘intervention modelling experiment’ (IME) for the PRIME approach). A multi-database searching strategy was adopted. Boolean operators were used in order to maximise the penetration of terms searched, while appropriate ‘wild cards’ were adopted to account for plurals, variations in databases and spelling. Reference lists of relevant publications and key journals were hand-searched.

Because of the broad nature of scoping reviews, databases were searched at different points in time, and results were used to inform the following searches in order to get a balance between breadth of the search and relevant hits. Appendix 1 shows examples of search strings used together with the decision-making process that led to the constant refinement of the search strategy, in order to ensure the coverage of the most relevant literature.

The variation of terminology used in the field and the identification of the most appropriate keywords represent one of the main challenges we faced in this review. It transpired that different terms could have been used to refer to an optimisation study and thus we conclude that there is a substantial lack of consensus on the terminology and core concepts related to ‘optimisation’. Thus, for the purpose of this scoping review, not only did we search for papers that specifically used the word optimisation, as this would have led to the identification of only a subset of all possible studies. Moreover, this review used a search strategy developed through the different stages of the review process, which combined keywords related to the overall process of optimising complex interventions together with framework or guideline-specific keywords used to describe an optimisation process (Appendix 1). In this way, we aimed to identify a heterogeneous group of studies that optimised the intervention under development prior to the full-scale RCT by following different frameworks and guidelines.

### Study selection

One author (SL) screened all of the titles and removed any obviously irrelevant records, such as studies from other fields (e.g. biomedical and pharmaceutical). One reviewer (SL) assessed all abstracts for relevance. To check for rater reliability, a second review author (RF) independently assessed a randomised subset of 15 % of abstracts and the full text of all papers for which there was uncertainty about inclusion. Disagreement was resolved by consensus between SL and RF, with input from a third reviewer (SH) where necessary.

### Extraction of results

Data were extracted from the included studies using a structured form. We systematically extracted information relating to study characteristics (author, date of publication and country of the study), intervention under development, framework used to guide the development stage, areas of focus of the optimisation process and the methods used. Although there is strong emphasis in the literature on the need for clear objectives for any study, such as pilot and feasibility studies [20], not all the included studies clearly reported the objectives of the optimisation studies conducted. However, it was possible to identify an overall area of interest for each reported use of an optimisation process. Thus, for the purpose of this paper, the term *areas of focus* was used to identify the different objectives and more generally the areas addressed by each optimisation process included [21]. In the case of missing data, attempts to contact the corresponding author of the study were made by SL. The data extraction process and form were initially piloted by two review authors (SL and RF) on five papers. Each author then independently extracted data from the remainder of the studies.

### Data analysis

We used the hierarchical task analysis (HTA) approach to explore the mechanisms and the structure that characterised optimisation processes within each individual study. Hierarchical task analysis, developed in 1971 by Annett, is an engineering and decision analysis-based process for decomposing and describing an activity, which can be used to analyse any type of task in any domain [22]. A key feature of HTA is that tasks—what people are seeking to achieve—are defined by goals. Thus, complex tasks, such as optimisation studies, can be analysed by deconstructing a hierarchy of goals, sub-goals and activities with a parent–child relationship at each level in the hierarchy [23]. In particular, each individual study was decomposed according to (1) the aim and the area of focus of the optimisation process, (2) the methods adopted and (3) the outcome of the optimisation process.

Graphical representations of each study flow were subsequently analysed and compared, in order to compare the tasks involved and the structure of different optimisation studies. Following this strategy, we were able to identify and explore similarities and differences across various optimisation processes for all included studies.

## Results

### Studies identified

**Fig. 3:**
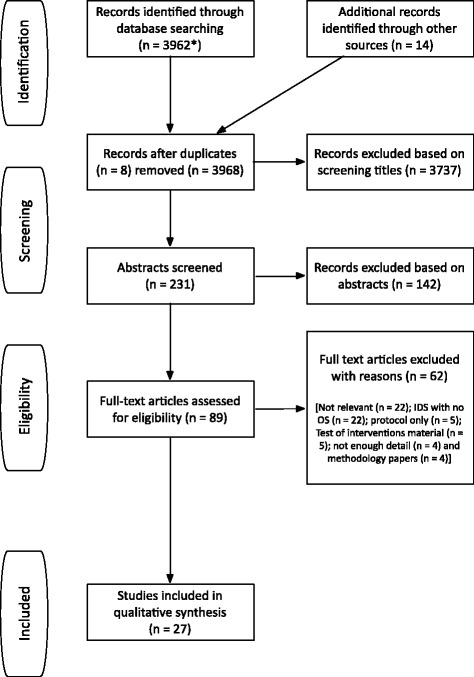
Study flow diagram. (In *asterisk*) Duplicates between databases searched simultaneously were removed automatically, whereas further duplicates were retrieved manually

After removal of duplicates, we screened the titles of 3968 papers. We identified 231 potentially relevant studies and, after abstract screening, 89 full papers were considered for inclusion in the review. Twenty-seven studies were finally included. Results of the search are displayed in Fig. 3. Table 3 provides an overview of the intervention, geographical location, framework, methods and area of focus of the optimisation process for each included study.

**Table 3:** Characteristics of included studies

Study	Country	Intervention	Framework adopted	Focus of the optimisation process	Methods	Outcome
Barley et al. 2012 [30]	UK	Nurse-led intervention to improve mood and cardiac outcomes in depressed coronary health disease patients	MRC framework 2008	Determine potential acceptability of the intervention to patients (in terms of method of delivery, duration and components), identify changes needed in relation to intervention components	Focus groups with patients (*n* = 13) and further evidence review to confirm/modify intervention components	The intervention was tested further in a feasibility study
Burr et al. 2011 [33]	UK	Glaucoma screening intervention	MRC framework 2008	Determine feasibility and acceptability to eye care providers, policy makers and health service commissioners of a range of intervention combinations and relative cost-effectiveness	Semi-structured interviews with eye care providers (*n* = 25), nurses (*n* = 5), GPs (*n* = 4), healthcare service commissioners and policy makers (*n* = 9), economic modelling	The integration of findings allowed to isolate a short list of candidate components that could be implemented in a RCT
Byng and Jones 2004 [40]	UK	Mental Health Link, an intervention to improve shared care for patients with long-term mental illness	MRC framework 2000	Critique the proposed intervention and evaluate practitioners’ perceptions of the different components of the intervention	Multi-method iterative design, including focus groups with a group of mixed health care professionals (*n* = 10) and a group of local experts in primary mental health (*n* = 6), semi-structured questionnaires and in-depth interviews	The intervention and the core components were refined and details added before this was piloted in three practices
Carnes et al. 2013 [25]	UK	Self-management intervention for chronic pain patients	MRC framework 2008	Test the feasibility of delivering the intervention and the receipt of the intervention	Uncontrolled pilot study, including observations, feedback questionnaires to course facilitators and participants, and participants interviews (*n* = 13)	Findings supported the development of a definitive RCT
Clyne et al. 2013 [26]	Ireland	OPTI-SCRIPT intervention to decrease potentially inappropriate prescribing (PIP) in older people	MRC framework 2000 and 2008	Testing of the components of the intervention with GPs (assessing GP perspectives on intervention and testing of the intervention	Focus groups and interviews with GPs	Intervention components were refined and a third component identified. Findings from the pilot study allowed further refinement to produce the finalised
Collins et al. 2005 [10]	USA	Smoking cessation intervention	MOST framework	Identify the most effective set of intervention components	16-cell fractional factorial experiments to test six components and their combinations	Phase to be completed^a^
Collins et al. 2011 [44]	USA	Smoking cessation intervention	MOST framework	Identify the most effective components or combination of components and the appropriate dosage levels	Fractional factorial design with 32 experimental conditions to test six intervention components	Phase to be completed^a^
Eldridge et al. 2005 [50]	UK	Intervention to prevent fall-related injuries in older people	*Not specified*	Estimate intervention effectiveness and long-term impact	Probability analysis for the effectiveness of the intervention, Markov model of long-term effectiveness and cost-effectiveness of the intervention	Results suggested that the intervention would be minimally effective. The intervention did not move to the full-scale RCT
Ettema et al. 2014 [24]	Netherlands	The Prevention of Decline in Older Cardiac Surgery Patients (PREDOCS) programme, a nursing intervention to prepare frail older patients for cardiac surgery	MRC framework 2008	Delineate intervention components, identify how components could be interrelated and identify how components relate to the outcomes	Comparison of evidence on effective interventions derived from the systematic review with the valuable theoretical understanding of the likely process of change obtain from the analytical study; survey study amongst nurses (*n* = 250), semi-structured interviews with patients	Findings led to the development of the intervention that was then tested for face validity
Farquhar et al. 2011 [34]	UK	Palliative Breathlessness Intervention Service (BIS) in patients with intractable dyspnoea	MRC framework 2000 and 2008	Explore participants’ experience of using the intervention and clinicians’ experience of referring patients to the service	Interviews with patients and patients’ relatives (*n* = 10), clinicians (*n* = 4) who had use the drafted service	The intervention was remodelled on the basis of the findings and then piloted
Booth et al. 2006[52]						
Grant et al. 2014 [48]	UK	Data-driven quality improvement in primary care (DQIP) intervention to improve prescribing safety in primary care	*Not reported*	Explore GPs and practice managers’ perception of the value of the specific components of the intervention (education, informatics and financial), their experience of adopting and implementing the intervention into routine practice and to changing prescribing behaviour	Semi-structured interviews with GPs and practice managers	Practice experiences identified some barriers which facilitated optimising the intervention beyond suggestions in the literature on changing prescribing
Gray et al. 2013 [27]	UK	The Football Fans in Training (FFIT) weight loss intervention for adult men	MRC framework 2008	Gain feedback from participants and coaches on the programme and its delivery	Observations, interviews and focus groups with participants and coaches and questionnaire to participants	The intervention was refined and then tested in a full-scale RCT
Hrisos et al. 2008 [46]	UK	Two behavioural interventions to promote GP management of upper respiratory tract infection (URTI) without prescribing antibiotics	Intervention modelling experiment	Explore the potential effect of the intervention on proxy outcomes that represent the actual behaviour	2 × 2 factorial randomised controlled trial involving 340 GPs. GPs were asked to complete two postal questionnaire surveys which included clinical scenarios	Findings encouraged the development of a replicable methodology for the design, evaluation and refinement of interventions prior to service-level RCTs
Kirkevold et al. 2012 [31]	Norway	nursing intervention for psychological health and well-being after stroke	MRC framework 2000 and 2008	Evaluate and critique the intervention by patient and relative representatives, clinical experts and researchers	Consensus process involving a wide range of healthcare professionals patients representatives and family carers	The intervention was refined and then tested in a feasibility study
Lewis et al. 2013 [28]	UK	Guided self-help (GSH) intervention for the treatment of mild-to-moderate post-traumatic stress disorder (PTSD)	MRC framework 2000 and 2008	Test the intervention potential effect and stakeholders’ perspective in order to refine the intervention	2 small-scale and uncontrolled pilot studies (*n* = 10 and *n* = 9) including pre and post treatment quantitative data, interviews with participants taking part in the pilots and focus groups with healthcare professionals and stakeholders involved in the development of the prototype	Findings from the first pilot led to the development of the intervention prototype, which was then tested in a second pilot. Results were used to refine the programme in order to produce the finalised programme
Lovell et al. 2008 [35]	UK	Guided self-help intervention for depression in primary care	MRC framework 2000	Synthesise available evidence on the effectiveness of the intervention, identify key factors that may moderate effectiveness and deal with uncertainties emerging from the reviews, assess of acceptability to patients and healthcare professionals	Meta-regression, meta-analysis and a consensus process with experts, including academics (*n* = 8), health professionals (*n* = 10) and service users (*n* = 1), phone interviews with patients and healthcare professionals	The integration of findings allowed identifying the ‘core components’ of the intervention, which was then tested in feasibility study. The intervention did not moved to the RCT phase, as it did not markedly improve outcomes in the exploratory study
Munir et al. 2013 [42]	UK	Work-related guidance tool for people with/recovering from cancer	Intervention mapping [36]	Obtain consensus on the questions included in the guidance tool (a list of 43 questions was previously developed) and to which healthcare professional these should be asked, and test feasibility of the intervention to participants	A two-round Delphi study conducted online with 172 experts (round 1) and 139 experts (round 2); online survey to participants (*n* = 38) who tested the guideline tool for six weeks	The intervention was finalised by identifying the key components of the tool and a range of stakeholders and the tested in a feasibility study
Murchie et al. 2007 [37]	UK	Follow-up programme for people treated for cutaneous malignant melanoma	MRC framework 2000	Seek patients and GPs’ views on feasibility, desirability, benefits and components of the programme; assess feasibility and identify problems or deficiencies	Steering group consultations, semi-structured interviews with patients (*n* = 9) and GPs (*n* = 14), pre-pilot operationalisation exercise	The components of the intervention were identified, fine-tuned and the final intervention tested in a feasibility study
Palmer et al. 2013 [29]	UK	Nurse-led intervention for the outpatient management of incidentally diagnosed pulmonary embolism in cancer patients	MRC framework 2008	Real-time re-modelling, refinement and optimisation of the intervention to respond to problems and deviations arising in practice	Observations, audit and survey	The intervention processes and delivery were refined on the basis of the real-time re-modelling process’ results
Redfern et al. 2008 [36]	UK	The Stop Stroke intervention to improve risk factor management after stroke	MRC framework 2000	Achieve consensus about the factors which a novel intervention should address and how this might be delivered and test feasibility of intervention components to patients and healthcare professionals	Consensus process involving a study steering group (a team of multidisciplinary experts) and local clinicians, researchers and stroke survivors, semi-structure interviews with patients and healthcare professionals as part of the feasibility trial	The intervention components were identified, then the intervention was tested in a cluster RCT and parallel process evaluation
Robinson et al. 2005 [39]	UK	Intervention to facilitate coping skills in new carers of stroke patients	MRC framework 2000	Explore carers’ experiences of caring and their views on the proposed theoretical intervention, including the content, appropriateness and delivery of the intervention	One-to-one interviews with carers (*n* = 14), focus group with a subset of participants, interviews with carers that participated in the first course (*n* = 5), satisfaction questionnaire (*n* = 7)	Following analysis of the qualitative data, the theoretical outline of the course was refined
Schaefer et al. 2013 [49]	USA	Smoking cessation interventions	*Not reported*	Use simulations to estimate potential changes on the outcomes of interest and evaluate alternative intervention scenarios	Simulation algorithms to test peer influence on smoking and smoker popularity	Results demonstrated the potential impact of the behavioural intervention
Smith et al. 2012 [32]	UK	Intervention to reduce time to presentation with lung cancer symptoms	MRC framework 2008	Explore the theoretical intervention with GPs and patients	Interviews with lung cancer patients (*n* = 6), focus groups with people with lung cancer (*n* = 7), focus groups with GPs (*n* = 4), operational meetings with 2 GP practices	The study used the data from the focus groups and operational meetings to refine the intervention
Sturt et al. 2006 [38]	UK	The Self-Efficacy Goal Achievement (SEGA) nursing intervention for type 2 diabetes	MRC framework 2000	Explore intervention effectiveness, patients and healthcare professionals perspective of the intervention and establish the validity of any resulting changes to the intervention	Small uncontrolled trial of the intervention with 2 practice nurses and 8 participants (from 2 practices) and scenario testing	The intervention was adjusted to remove the less effective components and enhance the more effective ones
Treweek et al. 2014 [45]	UK	Interventions to reduce inappropriate prescribing by general practitioners of antibiotics for upper respiratory tract infections	Intervention modelling experiment	Explore and refine theory-based interventions before moving to a full-scale trial by evaluating the potential effect of the intervention on proxy outcomes that represent the actual behaviour	Exploratory RCT involving 270 GPs. GPs were asked to complete web-based clinical scenarios and questionnaire	Findings supported the use of intervention modelling experiments to reduce iterations of full-scale trial/analysis/revision before an optimised intervention is produced
Vonk Noordegraaf et al. 2012 [43]	Netherlands	eHealth intervention for the empowerment of gynaecological patients during the perioperative period to obtain timely return to work (RTW) and prevent work disability	Intervention Mapping	evaluate whether the intervention fitted the expectations of healthcare professionals and patients	Evaluation questionnaires completed by patients (*n* = 15), physicians (*n* = 11), eHealth specialists (*n* = 3) and patient representative (*n* = 1)	Minor adjustments were made on the basis of the findings
Whittaker et al. 2012 [47]	New Zealand	Mobile health intervention to prevent the onset of depression in adolescents (MEMO)	Process for the development and testing of mobile phone-based health interventions	Determine acceptability of the proposed intervention to target audience, improve and refine intervention on the basis of feedback	Online surveys involving students (*n* = 40), focus groups and individual interviews with adolescents	Results informed the development of the intervention

^a^Data not available at the time of this study was completed

Most of these studies were conducted in Europe and specifically in the UK (*n* = 19, 70.4 %), the Netherlands (*n* = 2, 7.4 %), Ireland (*n* = 1, 3.7 %) and Norway (*n* = 1, 3.7 %). Three studies were conducted in the USA (11.1 %), and one study was conducted in New Zealand (3.7 %). The majority of included studies were published from 2011 onwards (*n* = 17, 63.0 %) (Table 3).

### Guidelines or frameworks used to guide the intervention development process

A range of different guidelines or frameworks for the development and evaluation of CHIs were employed by authors of the included studies. In particular, 17 of the 27 studies included in this review used the MRC 2000 framework, the updated 2008 version or a combination of the two [24–40]. Two studies adopted the intervention mapping framework [41] developed by Bartholomew and colleagues in 1998 [42, 43] and two applied the MOST framework [10, 44]. In addition, two studies followed the intervention modelling process [45, 46] and one study conducted in New Zealand introduced and applied new guidelines specifically for the development of mobile health interventions [47]. The remaining studies did not specify the guidelines or frameworks adopted to develop the intervention of interest [48–50].

### Types of intervention

The interventions reported across the review varied widely and included a few targeting behavioural change at the individual patient level, such as weight reduction [27] and smoking cessation programs [10, 44, 49], or at the level of healthcare professionals, such as interventions targeting general practitioners to reduce inappropriate prescribing behaviours [26, 45, 46, 48]. Interventions were delivered in a variety of settings (e.g. inpatient, outpatient clinics and home-based settings) and targeted a wide range of conditions, such as mental health conditions [28, 30, 35, 40, 47], stroke [31, 36, 39], cancer [29, 32, 33, 37, 42] and other chronic illnesses [25, 34, 38]. Two studies reported on preventive interventions targeting older people [24, 50] and one on a programme to empower patients undergoing gynaecological surgery during the perioperative period [43].

**Fig. 4:**
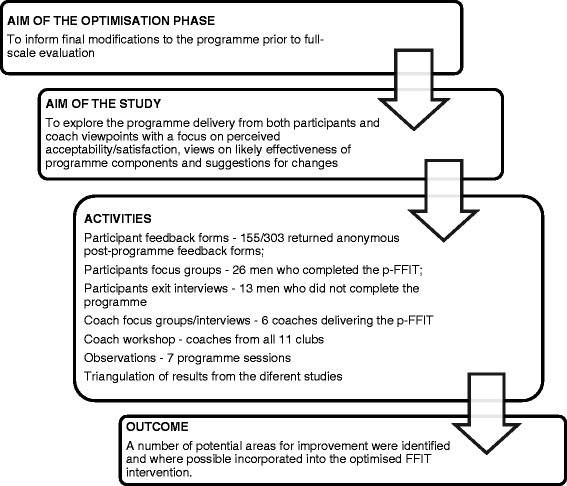
Representative examples of the HTA applied to Gray et al. [27]. Gray et al. [27] reported on the development and optimisation of the Football Fans in Training programme (p-FFIT), an intervention to help men lose weight, become more active and adopt healthier eating habits. In the programme development phase, an expert multidisciplinary group led the development of the pilot programme. The aim of the optimisation phase was to inform final modifications to the programme prior to full-scale evaluation. This phase involved a series of qualitative studies to explore the programme delivery from the viewpoints of both participants and coaches. These include participant feedback forms, participant focus groups, telephone or face-to-face interviews with non-completers, coach focus groups and interviews, a coach workshop and programme session observations. The coding frame was based on perceived acceptability/satisfaction, views on likely effectiveness of programme components and suggestions for changes. The process evaluation confirmed that the p-FFIT was highly acceptable to both participants and coaches. A number of potential areas for improvement were identified and, where possible, incorporated into the optimised FFIT intervention

Figure 4 shows a representative example of how we applied the HTA approach to one of the included studies [27]. Results emerging from the comparisons of the different tasks are described under the following main conceptual categories: areas of focus, methods used and outcome of the optimisation processes.

### Areas of focus of optimisation processes

The common aim of all included studies was to evaluate or test health interventions or their components before moving to a definitive trial. However, closer inspection using the HTA approach showed that they focused on a series of different aspects of the intervention under development.

Fourteen studies explored the acceptability and/or feasibility of the intervention to different stakeholders, including providers, recipients or policy makers and service commissioners [27, 29–32, 34, 36, 37, 39, 40, 42, 43, 47, 48]. Seven studies were a preliminary investigation of the effect of the intervention or a combination of its components on proxy or final outcomes and intervention long-term impact [10, 24, 44–46, 49, 50]. Six studies evaluated more than one element; in particular, they explored the acceptability to different stakeholders together with either the intervention’s effectiveness [25, 26, 28, 35, 38] or cost-effectiveness [33].

### Methods adopted

A number of different methodological approaches were used to undertake the optimisation process (Table 3). Several studies employed well-established qualitative methods including interviews or focus groups with providers and recipients or relevant stakeholders [26, 30, 32, 34, 39, 47, 48] and observations and consensus processes including researchers, patients and clinical experts [31]. A number of studies adopted quantitative methods such as fractional factorial experiments [10, 44], economic modelling [33], small uncontrolled pilot studies (with no control group) [25, 29, 38], experimental 2 × 2 randomised controlled trials [45, 46], probability models [49, 50] and evaluation questionnaires [43]. Several studies used mixed methods, combining qualitative strategies to explore stakeholders’ perspectives and quantitative analysis to estimate the intervention effect [24, 27, 28, 33, 35–37, 40, 42]. The level of detail reported on the methodology used varied widely across the studies (Table 3).

Overall, the use of specific methods appears to be informed by the research questions and the areas of focus of the single optimisation study. For example, factorial experiments, uncontrolled pilot studies and probability models tended to be used to test specific components and to identify an intervention’s potential effect [10, 25, 29, 38, 44, 49, 50]. Optimisation strategies applying qualitative methods tended to explore acceptability and feasibility issues [26, 30, 32, 34, 39, 47, 48]. On close inspection, it was possible to classify optimisation strategies in relation to *when* the optimisation process takes place with reference to the pilot stage. In particular, *prospective* strategies tend to look at theoretical feasibility and acceptability issues and are completed before the pilot stage; as in the optimisation studies conducted by Barley [30] and Byng and Jones [47], where focus groups with patients and healthcare professionals were run to explore the potential acceptability and feasibility of the proposed intervention to patients and practitioners. In vivo strategies, such as the real-time re-modelling and optimisation approach applied by Palmer and colleagues [29], explore actual implementation issues, by allowing researchers to immediately respond to problems and deviations arising in practice in a pilot setting. The last category, the *retrospective* strategies tend to explore stakeholders’ feedback once they have piloted the drafted intervention; thus, as in the optimisation study conducted by Lewis [28], a group of patients affected by post-traumatic stress disorder who took part in the piloting of the drafted intervention were interviewed to explore their perspectives on the intervention and its components, in order to identify potential required changes.

The included studies reported on the involvement of different stakeholders, who can be grouped as follows: service users, such as patients, informal carers and family members; service providers, including GPs, nurses and other healthcare professionals; and ‘other’ stakeholders, including academics and researchers, organisation representatives and policy makers. Of note, there were conflicting reports about the added value of stakeholders in optimisation studies. The involvement of stakeholders was identified as a strength by several studies, as stakeholders play a key role in the implementation of interventions into practice [30, 32, 33, 42, 43, 45]. However, other studies suggested that the process of identifying stakeholders and the potential lack of representativeness of the stakeholders involved were a limitation for optimisation studies [27, 37].

### Outcome of the optimisation processes

Overall, the optimisation processes implemented across the studies included in this review were viewed positively by the authors; providing researchers with important information about the potential effectiveness of the intervention and informing decisions on how (or whether) to proceed to the next stage. The majority of studies (*n* = 16) subsequently refined the intervention to then test the optimised version of the intervention within pilot studies or full-scale RCTs [24–32, 34, 38–40, 43, 47, 48]. In two studies, findings from optimisation processes discouraged researchers from moving to the full-scale RCT stage, as the interventions did not show the potential for effectiveness that was expected [35, 50]. A few studies reported that the optimisation processes allowed the research team to identify the ‘best ingredients’ out of several candidate components [33, 35–37, 42]. Despite attempts to contact the leading authors, data on the outcome of two of the included optimisation studies were not available [10, 44].

## Discussion

To the best of our knowledge, this is the first review to synthesise the different strategies used to optimise CHIs prior to full-scale RCTs. Empirical studies were identified to map current practice and identify gaps in the literature on intervention optimisation. Interestingly, most studies identified were published within the last five years, suggesting a recent surge in interest from the research community in maximising, up-front, the potential effectiveness of CHIs and reporting pre-trial evaluation processes. This is in line with the consensus that pre-trial stages are increasingly seen as critical steps in the development of new interventions, as sub-optimal intervention design may result in weak effects [9].

The novel use of the HTA method to facilitate decomposition of optimisation studies allowed us to isolate (a) factors that are currently prioritised and tested prior to evaluation by RCT, (b) the methods used in optimisation and (c) the outcome of each optimisation process. The areas of focus of the different optimisation studies included the acceptability and feasibility of the intervention to key stakeholders but also the exploration of the potential effects and cost-effectiveness of the intervention. This suggests that the acceptability of the intervention to those directly involved in the delivery and receipt of the final intervention, together with the anticipated effect of the intervention, are important elements to take into account as early as possible in the pre-trial stage. Results also indicate that the area of focus of the optimisation process informed decisions on which methods to use. For example, economic modelling and probability models were used to explore costs and the potential effectiveness of interventions, whereas interviews and questionnaires were generally used to evaluate acceptability to service clients and healthcare professionals.

From an in-depth analysis of the included studies, we were able to classify optimisation strategies as prospective, in vivo and retrospective. It is possible to argue that prospective strategies tend to explore theoretical perspectives and hypothetical intentions from different stakeholders in relation to the proposed intervention, presented through oral or video presentations and informative material. On the other hand, retrospective strategies look for stakeholders’ feedback and involve people (such as service clients and providers) who have piloted the intervention in a small, often uncontrolled, study. In vivo strategies, instead, look at implementation issues, in order to identify and apply potential changes to the draft intervention. When retrospective and in vivo optimisation strategies are applied, stakeholders are involved in the piloting of the drafted intervention; thus, their feedback on feasibility and acceptability is informed by a ‘real’ experience of the intervention. Whereas, prospective strategies allow stakeholders to influence from the outset rather than once the piloting is underway. This suggests that different strategies can be applied in different situations and for different purposes; for example, prospective strategies might be more appropriate in the design and development of the intervention, to help researchers identifying those components that increase the feasibility and acceptability of the intervention to the groups of people directly involved. Retrospective strategies might be helpful to gain confirmation of the potential effect of the intervention and its potential feasibility. Finally, in vivo strategies might be used in those situations where researchers are looking to implement changes during the pilot process to immediately verify how these influence the intervention effect.

Given the current financial constraints upon health services research and the large number of trials that fail to show effectiveness, it has been suggested that it is increasingly important to define strategies that support researchers in the development of more effective interventions [3, 50, 51]. This review evidences the emerging role of optimisation studies in developing interventions that are potentially more likely to be effective and highlights that a range of strategies are used for a range of different purposes and that greater clarity in both the terminology and the selection of different methods in order to develop and improve interventions would be helpful.

Some of the studies included suggested that optimisation has the potential to support researchers in identifying interventions or components that are likely to fail or show little effect if implemented in a full-scale RCT, but questions as to when the intervention is *ready* to be evaluated in a realistic setting and *how* researchers decide whether to move to a full-scale RCT still need to be answered. As Sermeus [9] suggests, complex interventions indeed generate some effect, but ‘the real question is how to establish when this is enough’ and when the intervention has been optimised enough to be the best intervention possible.

We believe the findings and the questions emerging from this scoping review should inform future research exploring the mechanisms of actions and the benefits and challenges of conducting optimisation studies. This review could also be used to generate much needed discussion amongst healthcare researchers undertaking complex intervention trials on when to apply different methods and which are most useful in relation to different circumstances and types of intervention. Furthermore, findings from this review could help researchers in thinking about and planning future optimisation studies, which could ultimately lead to the design of more successful RCTs.

### Strengths and limitations

To our knowledge, this is the first review to document the literature available on pre-trial strategies for the optimisation of CHIs. The HTA analytical approach enabled a detailed analysis of the different tasks and tools involved in different optimisation strategies, which led to the development of a preliminary classification of optimisation strategies. Furthermore, the iterative nature of the scoping review allowed us to extensively explore the literature available on the topic of enquiry; however, by its nature and the issues related to the terminology currently in use, it does not aim to be exhaustive. It is therefore possible that other optimisation strategies exist, which have not been captured by this review.

### Implication for research

Future research should explore, in-depth, the decision-making process behind optimisation studies, the benefits and challenges of optimising CHIs and those related to specific optimisation strategies, by gaining researchers’ accounts of the process of optimising complex interventions. Furthermore, examples of RCTs of complex interventions should be investigated in order to explore the impact of optimisation processes on the effectiveness of the intervention implemented in a real-life setting.

## Conclusions

In summary, our review explored strategies and methods that are currently used prior to a definitive RCT to assess situations of sub-optimal intervention design and to anticipate potential implementation failure. Findings from this scoping review represent the first step towards helping healthcare researchers to plan and conduct studies aimed at identifying what works and what does not work within the intervention under design, in order to ensure that those interventions and intervention components which proceed to full-scale RCT are those most likely to be effective. The review suggests a classification of optimisation strategies which is of help in understanding which methods to use in different situations, but it also raises a series of questions in relation to how researchers know when the intervention is ready or optimised enough to move to the full-scale trial stage. In the current economic climate, the answers to these questions are deemed invaluable in fostering a wiser use of public funding for the development and evaluation of more effective interventions.

## Appendix 1

**Table 4:** Examples of iterative searches conducted to identify relevant studies

Searches	Decision-making process
1. Searches conducted: on the first week of June 2013	The first round of searches looked for studies that aimed to develop CHIs. After completing these searches the team met to discuss the relevance of the hits identified. Results from the searches were too broad, therefore the team decided to narrow the searches to look for studies that reported on optimisation processes.
The Cochrane Methodology Register	
S1 complex intervention ti, ab	
S2 design ti, ab	
S3 development ti, ab	
S4 S2 OR S3 ti,ab	
S5 S1 AND S4 ti,ab	
MEDLINE, CINAHL, AMED	
S1 complex intervention ab	The studies identified through the Cochrane Methodology Register and AMED were not particularly relevant for the review. Therefore, the team excluded these databases from the following searches.
S2 health intervention ab	
S3 allied health intervention ab	
S4 S1 OR S2 OR S3	
S5 develop* ab	
S6 design* ab	
S7 S5 OR S6 ab	
S8 S4 AND S7 ab	
PsycINFO, PROQUEST Nursing and Allied Health Source	
S1 complex intervention ab	
S2 health intervention ab	
S3 allied health intervention ab	
S4 S1 OR S2 OR S3	
S5 develop* ab	
S6 design* ab	
S7 S5 OR S6 ab	
S8 S4 AND S7 ab	
2. Searches conducted: on the first week of September 2013	The second round of searches looked for pre-trial studies that had applied and reported on the process of optimising CHIs. Results highlighted issues in relation to the terminology used. In particular, the keywords related to ‘optimisation’ retrieved a series of studies related to other disciplines, such as biomedicine and statistics.
MEDLINE, CINAHL	
S1 complex intervention ab	
S2 health intervention ab	
S3 nursing intervention ab	
S4 S1 OR S2 OR S3	
S5 optimi?ation ab	
S6 optimising ab	
S7 S5 OR S6	
S8 S4 AND S7	
3. Searches conducted: on the last week of October 2013 and updated on the first week of March 2015	
MEDLINE, CINAHL	In the third round of searchers the team decided to include several keywords related to guidance and frameworks for the development and evaluation of CHIs. Therefore, these searchers looked for intervention development studies that either explicitly referred to optimisation or described processes that fitted with the definition of optimisation study used in this review.
S1 complex intervention ab	
S2 health intervention ab	
S3 allied health intervention ab	
S4 (MH “nursing interventions”) ab	
S5 S1 OR S2 OR S3 OR S4	
S6 optimi?ation ab	
S7 intervention modelling experiment* ab	
S8 modelling ab	
S9 phase 1 ab	This iterative process allowed to identify a search strategy that balanced between the breadth of the search and the relevance of the hit retrieved.
S10 S6 OR S7 OR S8 OR S9	
S11 S5 AND S10	
PsycINFO, PROQUEST Nursing and Allied Health Source	
S1 complex intervention ab	
S2 health intervention ab	
S3 allied health intervention ab	
S4 S1 OR S2 OR S3	
S5 optimisation ab	
S6 optimising ab	
S7 modelling ab	
S8 intervention modelling experiment* ab	
S9 S5 OR S6 OR S7 OR S8	
S10 S4 AND S9	

## Abbreviations

CHIs: complex health interventions
HTA: hierarchy task analysis
MOST: multiphase optimisation strategy
MRC: Medical Research Council
NPT: normalisation process theory
PRIME: process modelling in implementation research
RCT: randomised controlled trial
